# Maintenance of Cross-Sector Partnerships: The Role of Frames in Sustained Collaboration

**DOI:** 10.1007/s10551-018-3859-5

**Published:** 2018-04-25

**Authors:** Elizabeth J. Klitsie, Shahzad Ansari, Henk W. Volberda

**Affiliations:** 1 Department of Strategic Management and Entrepreneurship, Rotterdam School of Management, Erasmus University, 3000 DR Rotterdam, The Netherlands; 2 Judge Business School, University of Cambridge, Cambridge, CB2 1AG UK

**Keywords:** Cross-sector partnership, Frames, Framing, Mechanism, Collaboration, Maintenance

## Abstract

We examine the framing mechanisms used to maintain a cross-sector partnership (XSP) that was created to address a complex long-term social issue. We study the first 8 years of existence of an XSP that aims to create a market for recycled phosphorus, a nutrient that is critical to crop growth but whose natural reserves have dwindled significantly. Drawing on 27 interviews and over 3000 internal documents, we study the evolution of different frames used by diverse actors in an XSP. We demonstrate the role of framing in helping actors to avoid some of the common pitfalls for an XSP, such as debilitating conflict, and in creating sufficient common ground to sustain collaboration. As opposed to a commonly held assumption in the XSP literature, we find that collaboration in a partnership does not have to result in a unanimous agreement around a single or convergent frame regarding a contentious issue. Rather, successful collaboration between diverse partners can also be achieved by maintaining a productive tension between different frames through “optimal” frame plurality—not excessive frame variety that may prevent agreements from emerging, but the retention of a select few frames and the deletion of others toward achieving a narrowing frame bandwidth. One managerial implication is that resources need not be focussed on reaching a unanimous agreement among all partners on a single mega-frame vis-à-vis a contentious issue, but can instead be used to kindle a sense of unity in diversity that allows sufficient common ground to emerge, despite the variety of actors and their positions.

## Introduction

All the human and animal manure which the world wastes, if returned to the land, instead of being thrown into the sea, would suffice to nourish the world. (Victor Hugo, Les Misérables)

Most of us are not aware that the world’s food supply is seriously threatened by an approaching shortage of phosphorus reserves. This nutrient is a key ingredient in crop fertilizer that fuels high-yielding crops needed to feed the growing world population. Although scientists have sounded alarm bells about the impending phosphorus shortage in major news outlets such as *Nature* (Gilbert 2009; Kochian 2012) and *The Times* (Lewis 2008), this threat has not been given the attention it deserves. Several grassroots initiatives are, however, now trying to address this issue and raise awareness of it. In tandem, techniques are being developed to recycle phosphorus, rather than to mine it. Waste such as household trash and animal manure or even human manure can be used as input for phosphorus recycling. The Dutch Nutrient Platform is one of the initiatives aiming to address the “phosphorus challenge,” and takes the form of a cross-sector partnership (XSP), sometimes referred to as a CSSP (cross-sector social partnership). Within this platform, more than thirty partners are working together to create a market for recycled phosphorus. Yet initiatives such as this one are impeded by several technical and regulatory difficulties, as well as by extreme diversity among stakeholders. Despite these challenging conditions, the Nutrient Platform has nevertheless been able to coordinate the involvement of its diverse constituents and achieve significant regulatory reform. The platform took 3 years to establish, but has now been in full operation for the past 6 years, demonstrating its “capacity to create value” (Koschmann et al. 2012). This is a long time compared to the duration of many other similar XSPs. Indeed, being temporary in nature (Manning and Roessler 2014, p. 529), XSPs are vulnerable to derailment, failure or ineffectiveness (Le Ber and Branzei 2010; Poncelet 2001; Turcotte and Pasquero 2001). The Nutrient Platform has avoided this fate and continues to address the phosphorus challenge.

As an XSP, the Nutrient Platform is a multi-stakeholder collaborative initiative involving entities from different societal sectors—NGOs, government and private business—and is aimed at resolving the complex global environmental threat of an increasing shortage of phosphorus. While no one party is responsible for addressing the phosphorus challenge, if it remains unresolved, food security in the future will be in jeopardy. Taking on a complex challenge of this scale and scope requires collaboration across multiple organizations (Gray and Purdy 2018; Selsky and Parker 2005) and the development of “new organizational forms to accommodate the diversity of organizational activity taking place to address to social problems” (Crane 2010, p. 19). Cross-sector multi-stakeholder partnerships are one form in which such collaborations occur.

We aim to contribute to the literature on XSPs and multi-stakeholder collaborations by examining the dynamics underlying sustained collaboration. It is widely recognized that there are different stages of XSP development (Gray 1985, 1989): formation, implementation and outcomes (Selsky and Parker 2005). While many studies focus on the formation of XSPs (Koschmann et al. 2012; Manning and Roessler 2014) and their outcomes (Clarke and Fuller 2010; Clarke and MacDonald 2016), few studies examine how collaboration is sustained to allow XSPs to continue (e.g., Gray and Purdy 2018; Le Ber and Branzei 2010).

We examine how the various participants in the XSP both create an understanding of the issue in contention and negotiate conflicts between divergent interests. Responding to a call for more “longitudinal studies to investigate how XSPs evolve” (Selsky and Parker 2005, p. 86), we study how the framing work carried out by actors changes as a cross-sector partnership moves through different stages of evolution—variation, selection, deletion and retention (Campbell 1965; Lewin and Volberda 2003).

We take a framing approach to examining sustained collaboration in an XSP because “it is only at the micro level that the effects of institutions can be ‘directly’ observed” (Dacin et al. 2010, p. 1393). We draw on work on the micro-processes and mechanisms of framing, including the conceptual framework developed by Gray, Purdy and Ansari (2015), and identify specific mechanisms and tools used by cross-sector partners to sustain an effective collaboration. Whereas existing models of framing in collaboration often focus on convergence toward a single frame—through frame alignment, for example—our analysis suggests that maintaining optimal “frame plurality” (Gray et al. 2015) may provide a valuable way of understanding how agreements emerge. Maintaining optimal frame plurality requires parties to manage or tolerate multiple overlapping, conflicting, interstitial, or even unrelated meanings drawn from different sectors in the interest of getting work done (Gray et al. 2015; Kraatz and Block 2008). Our findings indicate that sustained collaboration can be achieved by creating a “productive tension” (Murray 2010) between different frames and maintaining “optimal” frame plurality—i.e., not using an excessive variety of frames but ret and recombining a few select frames and deletion others. We observed that frames evolve within a narrowing bandwidth as the collaboration progresses, and previous frames that have lost traction or no longer fit the discussion are discarded. This dramatically reduces the number of possible combinations, gets parties focused on the final aim and allows agreements to emerge. Furthermore, our data suggests that this frame evolution “occurs in a politicized social context and is inherently bi-directional” (Gray et al. 2015, p. 115), where changes in power arrangements arising from shifts in the partnership composition (such as more businesses entering the XSP) shape which frames are selected, discarded or retained.

By studying how collaboration may be sustained in an XSP, we contribute to the literature in three ways. *First*, studies have focused mostly on the formation of XSPs (Koschmann et al. 2012; Manning and Roessler 2014), their outcomes (Clarke and Fuller 2010; Clarke and MacDonald 2016), and different stages in their development (Gray 1989; Selsky and Parker 2005). We build on this work by shedding light on the process of XSP evolution, providing insights into some of the challenges that occur in XSPs after their formation and explaining how collaboration may be continued by sustaining optimal frame plurality amid the diversity of constituents and their differing positions regarding the issue at hand.

*Second*, our notion of optimal frame plurality extends related work that focuses on XSP dyads such as “frame fusion” (Le Ber and Branzei 2010) by examining how this complicated process takes place among a vast array of diverse partners from different sectors in an XSP which changes in composition over time. Also, while the concept of frame plurality has been theorized in previous studies (Gray et al. 2015; c.f., Murray 2010), our notion of optimal frame plurality suggests that plurality may have its limits and excessive variety may thwart sustained collaboration. We argue that the deletion of certain frames and the retention of a few may be necessary for generating optimal frame plurality and sustaining collaboration over time.

*Third*, while an impressive body of work on hybrid logics and hybridization has explained how plurality is managed in both organizational and inter-organizational settings by segmenting, bridging and recombining fragments of conflicting logics (e.g., Battilana and Dorado 2010; Battilana and Lee 2014; Pache and Santos 2013; Jarzabkowski et al. 2009; York et al. 2016), we show how actors manage this process of dealing with conflicting pressures together with other parties as a collective endeavor.

Next, we present a literature review on cross-sector partnerships, introduce our research context and case data and report our findings. We then derive a model for sustained collaboration and finally discuss some implications for future research.

## Cross-Sector Partnerships and Collaborations

Society faces a range of complex social problems or wicked problems (Rittel and Webber 1973) “defined by their circular causality, persistence, absence of well-structured alternative solutions, relative lack of room for trial and error learning” (Dorado and Ventresca 2013, p. 69; Reinecke and Ansari 2016). “The wickedness of the problem reflects the diversity of those involved in the issue” (Lach et al. 2005, p. 7). Addressing wicked problems requires collaboration between multiple organizations across sectors (Selsky and Parker 2005). Gray (1985, p. 912) defines collaboration as the pooling of resources, by two or more stakeholders, “to solve a set of problems which neither can solve individually.” This pooling occurs when problems are “complex, wide in scope, and beyond the scope of single organizations” (Westley and Vredenburg 1991, p. 67).

Though a collaborative multi-party effort is often necessary to address complex issues, the cooperation required can be very difficult, given the plurality and diversity of the actors involved (Gray and Purdy 2018). Indeed, fostering agreement among diverse parties over a contentious issue is highly challenging and fraught with obstacles. The new collaborative arrangement may include “formal or informal institutional arrangements of overlapping sectoral segments and/or combinations of governance mechanisms” (Seibel 2015, p. 697). While institutional pluralism has been recognized as a phenomenon at the field level, it can also occur at the organizational level (Kraatz and Block 2008), and entails “the co-existence of alternative, legitimate and potentially competing strategies within a single organization” (Jarzabkowski et al. 2009, p. 285; Reinecke and Ansari 2015). Murray (2010, p. 379) examines pluralism at the level of individual exchanges among actors and explains “the productive tension at the institutional boundary and the hybrids that emerge from it.” In her study of the patenting of a mouse engineered to study cancer, she explained how the differences between disparate parties do not necessarily dissolve for a collaborative arrangement to emerge, but rather may coexist productively. Similarly, work on hybrid organizations as “embodiments or incarnations of multiple logics” (Kraatz and Block 2008, p. 244) has provided rich insights into how organizations combine elements from different stakeholder domains and balance prescriptions from conflicting logics (e.g., Battilana and Lee 2014) and temporalities (Reinecke and Ansari 2015). Managing such diversity may be critical to sustaining XSPs.

### Types of Cross-Sector Partnerships (XSPs)

While collaboration across sectors is of interest to research in both public management (Bryson et al. 2015) and private management (Selsky and Parker 2005), the terminology used can vary. Waddell and Brown (1997: p. 1) use the term “intersectoral partnerships” to refer to collaboration between “organizations based in three sectors: the state (government), the market (business) and civil society (NGOs, non-profits, etc.).” Selsky and Parker (2005) discuss cross-sector social-oriented partnerships (CSSP) in which organizations from different sectors jointly address challenges. The main activities of XSPs include mutual problem-solving, information sharing and resource allocation.

In some ways, XSPs resemble alliances. Some scholars argue that the rationale for entering into a cross-sector collaboration is one of resource dependence (Selsky and Parker 2005, p. 851), where partners combine resources and skills to attain mutual benefits (Pfeffer and Salancik 1978). From this perspective, “partnerships present the opportunity to create a formidable, mutually reinforcing system which combines the unique capabilities and resources of each party to deliver outcomes beyond those of any one sector acting in isolation” (Googins and Rochlin 2000, p. 128). Studies using this approach tend to derive ideas from the more general alliance literature (Manning and Roessler 2014, p. 527). As in alliances, a common “interaction space” can ease communication and reduce uncertainty in XSPs (Ostanello and Tsoukias 1993). XSPs are formed in situations where “individual firms are one among many stakeholders whose activities are truly interdependent” (Gray 1985, p. 915). Collaboration thus involves “a cooperative, interorganizational relationship that is negotiated in an ongoing communicative process, and which relies on neither market nor hierarchical mechanisms of control” (Hardy et al. 2003, p. 323).

However, XSPs also differ from alliances in that cooperation to address wicked problems is often the key focus. As these problems are often deemed too large for a single organization or a sector to deal with, multi-party collaboration across sectors is often necessary (Westley and Vredenburg 1991). Hence XSPs are distinguished from “regular” alliances in that partner motivations are “a blend of self-interest and altruism” (Selsky and Parker 2005, p. 863). While XSPs are often designed as temporary projects, their aim is, however, to bring about *long*-*term* change (Manning and Roessler 2014, p. 528). While many studies of XSP formation assume that the mechanisms for general alliances also hold for XSPs, the boundary-spanning and project-based nature of XSPs distinguishes them from alliances (Manning and Roessler 2014). In addition, cross-sector partnerships face higher levels of complexity, due to the diversity of partners involved. Although some XSPs contain only two parties, these often come from different sectors and have conflicting core values (Nicholls and Huybrechts 2016), and unlike most alliances many XSPs are composed of multi-sectoral partners. These factors make XSP collaborations very complex to manage. As XSPs are often held together merely by the conviction of dissimilar actors about the key issue at hand (Selsky and Parker 2005, p. 863), they remain vulnerable to derailment or even dissolution.

#### XSP Life Cycle

Studies have looked at the life cycle of XSP evolution. Selsky and Parker (2005) distinguish between studies that focus on either XSP formation, implementation and outcomes. Waddell and Brown (1997) recognize five stages of XSP development: (1) identifying preconditions for XSPs, (2) convening actors and defining problems, (3) establishing a shared sense of direction, (4) implementing joint action strategies and (5) expanding and institutionalizing success. Research on XSPs often involves explaining one or more stages of the life cycle, particularly the formation stage. Le Ber and Branzei (2010, p. 184) take a framing approach to describe the formation of four XSPs. They conclude that collaborations move toward “frame fusion,” and once this is achieved, the new frame can be leveraged to address new emergent conflicts and problems. Koschmann et al. (2012) use a communication perspective to address XSP formation and propose a framework for the development of an “authoritative text” designed to create maximum value. With respect to outcomes, at least three broad categories are discussed in the literature: plan, process and partner outcomes (Clarke and Fuller 2010). In their study of how four Canadian community sustainability plans were implemented, Clarke and MacDonald (2016) draw on the resource-based view to situate XSP outcomes as collective resources that members gain from their involvement in these multi-stakeholder partnerships.

Despite the work that has been done on the formation and outcomes of XSPs, less attention has been given to the process by which they are sustained—even though maintaining collaboration between the various parties is a major challenge for partnerships of this kind. Once an XSP has been formed, it is of course hardly a given that it will attain its goals (Gray 1989; Huxham 1996), and many end in failure or become ineffective. Poncelet (2001) describes how in the EU Partnership for Environmental Cooperation (EUPEC), conflict between capitalist arguments and environmental concerns stifled progress and led to the maintenance of the status quo. The author concludes that a “non-confrontation practice” can stop a collaboration from “turning a critical eye toward some of the deeper, structural sources of current environmental dilemmas” (Poncelet 2001, p. 22) and prevent an XSP from bringing innovative solutions to complex social problems.

Turcotte and Pasquero (2001, p. 459) describe the case of waste management in Big City, where the diversity of partners meant that only objectives that were ambiguous could be agreed upon. The partnership thus “failed to produce what it had been designed for—a specific blueprint for an ecological waste management plan at the regional level.” Le Ber and Branzei (2010, p. 172) studied four cross-sector partnerships that sought to create social value. Although three of these collaborations were successful, one case—a partnership for minimally invasive surgery—was marked by continual conflict. Although the partnership contract was fulfilled in the end, the for-profit partner regarded the collaboration as a failure because little if any social value was created. Indeed, it is often a challenge for actors involved in XSPs to sustain collaboration and remain relevant and effective *after* an XSP has been formed. As many XSPs turn into “paper tigers” (e.g., Poncelet 2001; Turcotte and Pasquero 2001), it is important to examine what makes XSPs survive for longer periods of time and what will improve their chances of achieving their goals. Language and discourse, which have been identified as being important during the formation stage of XSPs (Westley and Vredenburg 1991, 1997; Koschmann et al. 2012), may also matter for collaborative partners in terms of helping them to maintain a collaboration beyond the formation stage. A key role is that of frames and framing.

#### The Role of Framing in Sustaining Collaboration

The XSP literature suggests that it is hardly a given that members of an XSP will continue to collaborate and sustain the partnership after its formation. While several factors influence XSP continuity, including the power configurations among the actors involved (Gray et al. 2015), one key communicative aspect of sustaining collaboration is framing—how actors skilfully use a variety of frames and rhetorical strategies to argue for their viewpoints and interests, as well as frames that emerge from their interactions. We follow Koschmann et al. (2012, pp. 333–334) in considering collaboration within an XSP to be the outcome of a “communication process (…) distinct from market or hierarchical mechanisms of control.” How issues are framed by different participants is central to this communication process.

Framing highlights certain aspects of a perceived reality in order to stimulate a particular understanding (Entman 1993, p. 52). Frames are “schemata of interpretation” that enable individuals “to locate, perceive, identify, and label” what happens in the world around them (Goffman 1974, p. 21). Frames define which actors are engaged, what kinds of problems are discussed, how these problems are defined, and what kinds of solutions are considered appropriate (Hoffman 1999; Lefsrud and Meyer 2012; Reinecke and Ansari 2016). Social movement scholars have shown how activists use collective action frames to “mobilize potential adherents and constituents, to garner bystander support, and to demobilize antagonists” (Snow and Benford 1988, p. 198). These collective action frames can involve “diagnostic framing” (problem identification and attributions), “prognostic framing” (possible solutions) and “motivational framing” (for collective action) (Snow and Benford 1988).

Other studies focus on the continuous negotiation that takes place, through ongoing interactions, to “reaffirm or challenge the frame repertoires available” (Gray et al. 2015, 116) in pursuing institutional maintenance and change. While a cognitive framing approach considers frames to be representations stored in memory, an interactional approach regards framing as “the dynamic enactment and shaping of meaning in ongoing interactions (and frames are transient communication structures)” (Dewulf et al. 2009, p. 162; Gray et al. 2015). Human behavior is thought to result from people drawing both on their existing frame repertoires and on frames that emerge during their interactions with others, as they use language and other symbols to create meaning in interactions (Cornelissen and Werner 2014).

While agreements may arise from the development of convergent positions among actors with different interpretations of the “truth,” they can also be reached by allowing a plurality of interpretations to coexist, and relying on “equifinal” meaning (Donnellon et al. 1986). This refers to agreeing about what action to take (e.g., collaboration) on a complex issue, despite disagreement between the different parties over why they may be doing this. For example, Reay and Hinings (2009) showed how different actors in healthcare teams used “pragmatic collaboration” to accomplish their work when faced with multiple and seemingly irreconcilable logics.

While frame plurality may explain the emergence of agreements in some cases, excessive variety in frames might arguably thwart these agreements and result in a failure to construct sufficient common ground to reach an agreement. At the same time, attempts to homogenize the frames of multiple and often disparate actors around a single convergent position may also breed conflict and lead to a failure to reach an agreement. Thus, both too much variety and too little variety may be unhelpful in achieving sustained collaboration. It is thus worthwhile to examine the evolutionary process through which certain frames are discarded or fall into disuse in an XSP, while others evolve, recombine and persist over time. To use evolutionary language (Campbell 1965; Lewin and Volberda 2003; Nelson and Winter 1982), it would be productive to examine the continuous cycle of variation, selection, retention and deletion of different frames over time in a multi-party partnership which spans sectors with differing organizational forms (businesses, government agencies and NGOs) and which seeks to address a complex global issue. This provides the main motivation for our research question: How do different frames used by multiple actors evolve over time and how might this sustain collaboration in a cross-sector partnership to address a complex global issue?

## Research Context, Design and Methods

To address the research question, we investigate how members of a Dutch XSP (the Nutrient Platform) used framing to maintain collaboration after the formation phase.

### Research Context

Phosphorus (P) is a chemical element, typically used as a main component of fertilizer. It is used to produce high-yielding crops deemed necessary to feed the growing world population (Kochian 2012). It is a non-renewable, non-substitutable resource (Lewis 2008) that organisms need as a component of DNA. In both humans and animals, phosphate is excreted through the digestive tract. It is thus present in human and animal manure, which can be used as a natural fertilizer. However, to create artificial fertilizers phosphorus is predominantly mined from mineral resources. These resources are finite and may not be sufficient for the world’s long-term needs (De Ridder et al. 2012). For the few countries where phosphate rock is found, it is fast becoming a strategic resource (Lewis 2008). Used phosphorus flows mainly into surface waters and poses a major threat to the environment. Phosphorus recycling is seen as the most promising way of addressing these issues (Gilbert 2009).

**Fig. 1:**
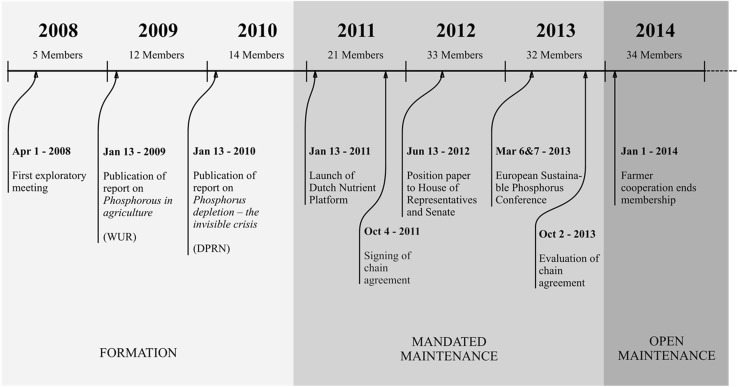
Timeline of main events in the Nutrient Platform

The Nutrient Platform (NP) is a Dutch multi-stakeholder group whose members “share a common concern for the global impact of phosphorus depletion and the way society is dealing with nutrients in general” (Nutrient Platform 2012). The Netherlands is one of very few countries with a phosphorus surplus. The reason for this is the large livestock sector. On April 1, 2008, the initial five members of the XSP had their first exploratory meeting. The aim was to create a market for recycled phosphorus. In 2009 and 2010, several key documents were published that catalyzed the growth in partnership membership. After more members had joined and an authoritative text had been drawn up’ (Koschmann et al. 2012), an official covenant was signed by 21 cross-sector partners and the Nutrient Platform was officially launched in 2011. In the covenant, each member noted their ambitions for the platform and pledged their (monetary or in kind) contribution to this shared purpose for an initial period of 2 years. After 2 years, this agreement was evaluated. The members then decided to continue the partnership to work toward further achievement of the goals set out in the initial agreement (see Fig. 1).

On January 1, 2015, the collaborative efforts resulted in a change in Dutch law, which allowed the previously prohibited trade of recycled phosphorus. By that time, the platform consisted of 35 diverse organizations, ranging from NGOs to engineering firms, government agencies, producers of (artificial) fertilizer and semi-public organizations. Though this significant achievement has taken the collaboration a step closer to its main goal, even in 2017 the partners are continuing to collaborate because their joint effort is still necessary to realize the ambitions that were outlined in the original covenant. The collective aim of the Nutrient Platform is to “close” the phosphorus cycle by recovering rather than discarding it after use. Nevertheless, individual organizational motivations for this goal range from social to financial. Some member organizations aim to achieve environmental sustainability, while others seek to make profits from recycling surplus phosphorus (from waste) into a valuable product that can be traded on the global market.

## Method

We draw on this case to understand a more general phenomenon, namely the use of framing to maintain collaboration in XSPs (Stake 1995). The case of the Nutrient Platform provides a fertile opportunity to study the maintenance of XSPs. With the signing of the 2011 covenant, the collaboration has successfully completed the formation stage in terms of agreeing on an authoritative text (Koschmann et al. 2012). Even though the initial covenant was for a period of 2 years, the partners still value the collaboration that has continued as new members join. The platform represents an example of a complicated cross-sector partnership with a relatively large number of partners, from all three sectors. The consensus in the XSP literature is that when the partners are more numerous, and less homogeneous in their organizational characteristics, it will likely be more difficult for “values to converge” across actors and organizations than when the stakeholders are fewer and more homogeneous (Selsky and Parker 2005, p. 864). This, in turn, may require more maintenance work to sustain the XSP.

In their work on strategy formulation and implementation in cross-sector partnerships, Clarke and Fuller (2010, p. 99) argue that more research should be conducted to investigate exactly how the number of partners changes the complexity of interactions within an XSP, and how this collaboration is implemented at the organizational level. We chose this particular platform because it enabled us to collect and analyze rich data and gave us access to the largely uncategorized archives relating to the collaboration. These provided a useful and relatively unbiased source of information, as the material they contained was in the main not geared to promoting the platform.

### Data Collection

This paper is based on data collected from the Nutrient Platform. Primarily, it draws on 27 interviews with members of the platform. These data are triangulated with over 3000 internal documents created within the platform during the period 2008–2015. These documents range from meeting agendas to strategic documents and marketing materials. Most of the documents are internally focused, though some—such as marketing materials and official communication materials—are aimed specifically at external audiences. The internal documents are especially insightful for examining conflicting views, while the externally aimed documents provide insights into some of the outcomes of these conflicts. We have reconstructed in detail the collaboration’s timeline from its inception in 2008 through to 2015. This data is supplemented by publicly available information about specific platform-related developments as well as broader socioeconomic developments that have affected the collaboration during the period from 2015 to 2017.

To define the platform and determine who the key actors were, two preliminary interviews were conducted with the current secretary [coordinator] of the Nutrient Platform as well as with the previous post holder. These two key informants were asked to describe which individuals and entities were either currently or previously involved in developing the platform. The interviews included all the actors who were characterized as “significant” or “long-term” players by the key informants and other interviewees. These included all the initial member organizations of the platform, including those who had since left, as well as their successors. The vast majority of representatives of organizations that continued to remain members for several years were also interviewed. Given their level of involvement, these significant players were expected to have the best recollection of past events. Nevertheless, all of their accounts were also cross-checked against each other and against the available internal documents. To avoid elite bias, in addition some individuals who were described by other interviewees as “new,” “former” or “inactive” members were also interviewed. “New” members were typically those who joined after 2011, when the initial agreement had already been signed. Former members were those who terminated their membership after 2011. “Inactive” members were official members of the platform who had no active involvement in the platform’s activities, other than attending (some) meetings. Only two people did not agree to be interviewed. One was a new member of the platform, and an interview was conducted instead with another new member. Another was a university researcher whose predecessor was interviewed instead.

To ensure comparability, the interviews were semi-structured. This allowed us to “obtain both retrospective and real-time accounts by those people experiencing the phenomenon of theoretical interest” (Gioia et al. 2013, p. 19). The questions focused on whether the interviewees agreed that there was a “phosphorus problem,” why they thought the platform existed, and whether the platform should be considered a success. A non-exhaustive list of questions asked during the interviews can be found in Table 1. The average length of the interviews was 45 min, with some lasting 1.5 h and others 20 min. All interviews were audio-recorded and transcribed verbatim.

**Table 1:** Overview of interview questions

Semi-structured interview questions
Why did you join the Nutrient Platform?
What are your activities for the platform?
What according to you is the reason that the platform exists?
Is the platform a success?
What are some successes of the NP?
What are some hurdles the NP has overcome or still should overcome?
Where is NP in 5 years’ time?
Would you characterize the platform as a social movement?

Archival data are used to complement the interview data and gain insights into the planning and execution of XSP activities. These data included internal documents from the Nutrient Platform—meeting agendas, minutes, strategic plans, memos, presentations, agreement signed when the platform was founded and evaluations of agreed upon targets. In addition, we observed member meetings of the Nutrient Platform and of the Steering Committee. Finally, for a period of 3 months, during which data collection took place, there was bi-weekly information exchange with the platform’s secretariat. Table 2 summarizes the different types of data collected. The table includes the number of interviewees per sector.

**Table 2:** Overview of data

Type of data	NP internal	NGO	Business	Knowledge institutes	Government
Interviews	Three interviews with current and past secretariat members	Three interviews	Twelve interviews, three of which were industry associations	Three interviews	Six interviews, one of which is with a member of the House of Representatives
Archival data	Full access was provided to all internal documents in the Nutrient Platform database, including meeting agendas, minutes, strategic plans, memos, presentations as well as the agreement signed to found the platform and several evaluations of the targets stated in that agreement				
Observations	One member meeting of the Nutrient Platform was observed as well as one meeting of the Nutrient Platform Steering Committee				

### Data Analysis

We first coded our data into incidents and categorized these into several event tracks (Van de Ven and Poole 1990). Figure 1 provides a summary of the main events.

We derived coding categories based partly on theoretical concepts such as framing mechanisms (Gray et al. 2015). We also coded for the different frames used by platform actors. Using NVivo10 software, we then refined these codes in more detail as we engaged with the data. The data points assigned to the theoretical coding categories were also coded according to the organization and individual where they originated in order to distinguish between frames and framing mechanisms used by actors from different sectors. Table 3 presents our resulting coding categories and verbatim examples of selected frames.

**Table 3:** Summary of main themes and verbatim examples

Theme	Sub-theme	Example
*Diagnostic framing*		
Crisis	1. Environmental impact—use of phosphorus	*The inappropriate use of phosphorus can (…) encourage the erosion and pollution of waterways, cause coastal dead zones and impact fisheries.* (Internal document, 2009)
	2. Environmental impact—mining of phosphorus	*As P supplies run down, we will need to use dirtier sources, leading to cadmium pollution of soil, and potentially even radiation pollution.* (Internal document, 2011)
	3. Scarcity	*The phosphorus problem is based on the idea that we have mines from where we get our phosphorus, so like oil and gas phosphorus is limited. If it is used up we will have a big problem.* (Interview with second Secretary of Nutrient Platform)
	4. Lack of security of supply	*Just five countries together control 90% of the world’s reserves of rock phosphate. China, the largest producer, has already begun to safeguard its supplies by imposing, in mid*-*2008, a 135% tariff on exports.* (NP press release, 2009)
Crisis + opportunity	5. Sanitation in developing countries	*Especially for developing countries it is interesting to create self*-*sustaining areas in terms of food security (for example by connecting cities (human excreta) to arable land for P*-*recycling) and energy (biogas).* (Internal document, 2010)
Opportunity	6. Availability of Dutch phosphorus	*As the only phosphorus surplus country in Europe, the Netherlands is in a unique position and faces special challenges. The present cost of manure processing is more than 100 million euros. Better defined and more effective composting and a wider range of recycling products can create alternatives for chemical fertilizer production and industry.* (Internal document, 2009)
	7. Dutch strengths in governance and innovation	*With the Phosphorus Chain Agreement the Netherlands has become a frontrunner in Europe, based on our knowledge and expertise in the agro*- *and food business.* (Public document produced by the Nutrient Platform 2012)

In addition, the frames identified were coded according to when they occurred in order to account for the possibility that frames would differ over time. Throughout our analysis, we identified three distinct phases of the XSP’s development. As per the existing literature, we demarcated a *formation* phase (Selsky and Parker 2005), during which the goals and organizing principles were being negotiated among the partners. This phase ended when an “authoritative text” was agreed upon (Koschmann et al. 2012). A 2-year period of *mandated maintenance* followed, during which an agreement of cooperation was signed by all parties involved in the XSP. We categorize as *open maintenance* a third phase that occurred after the agreed period had ended but when partners continued to cooperate. Although partners still referred to the original agreement, they were no longer bound by it in this phase. By cross-referencing the different frames that were employed by actors involved in the Nutrient Platform over time, we assessed frame *evolution.* Our analysis suggested an evolutionary process of variation, selection, retention and deletion when it comes to frames in active use. We then identified various mechanisms that enable this process.

## Findings

**Fig. 2:**
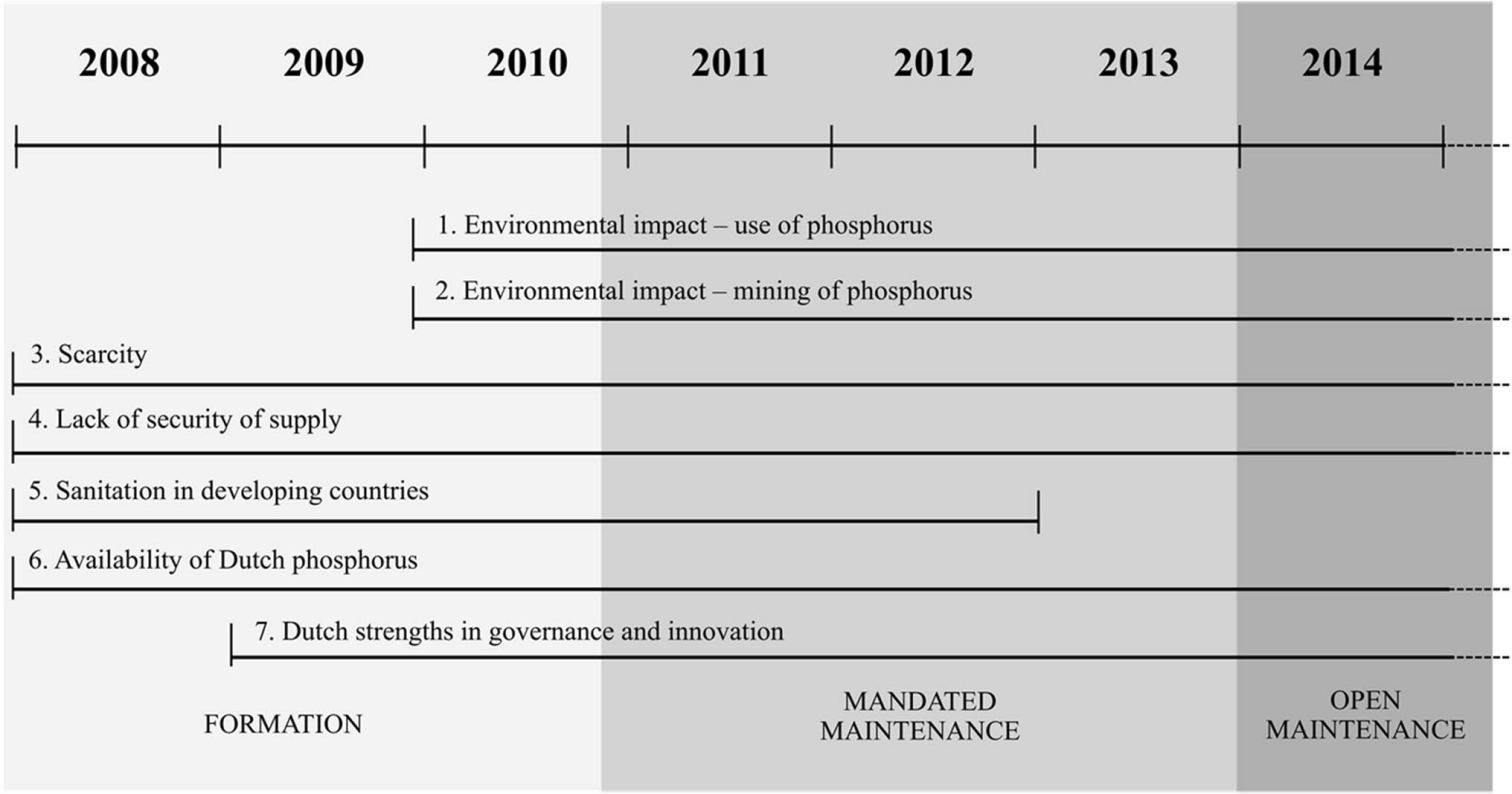
Active frames in different phases of the Nutrient Platform

A key finding of our study of the Nutrient Platform is that multiple frames were used in all life cycle stages of the XSP. We also found that in our three different stages of the platform life cycle (formation, mandated maintenance and open maintenance), different frames were used. Some frames were introduced after the collaboration had been in operation for several years, while one frame was selected out in the later stages (see Fig. 2).

When we examined this process, we identified factors that influence frame evolution. We found a wide variety of frames at the beginning but only a limited number of frames (and their re-combinations) survived over time (see Fig. 2).

### Multiple Frame Sources and Frame Variation

The frames used by those within the XSP vary because they originate from different sources. The difference is based on whether a frame was already present among members of the XSP, or whether it was later internalized. In the first category are frames that reflect the existing aims of XSP members. For example, the frame that views phosphorus availability as an economic opportunity for Dutch business (frame 6) has become a frame of the Nutrient Platform but was already in use in some of its member organizations:

I always said that transporting it [manure] from point A to point B within the Netherlands does not solve the problem of oversupply. We need to have less, Germany needs more, and Belgium needs more. There are other countries facing shortages. (VP for business development at a fertilizer company)

The alternative to frames that originate from internal members are frames that emerged based on the publication of information external to individual members. For example, one XSP member who is introducing a recycling alternative to mined phosphorus uses scientific information to support his argument that the current method of mining phosphorus is unsustainable:

The impurity of phosphorus is very important It contains uranium and cadmium. So, the phosphorus that is mined is strongly contaminated. This means that more radioactivity is brought on to the land when you use artificial fertilizer (CEO of a sludge-processing company)

This suggests that the environmental impact of mining (frame 2) is being used to underline that arguments for switching to phosphorus recycling are broader than simply a business case.

### Frame Selection

We found that of a very large number of frames resulting from frame variation, only a limited number were selected for active and repeated use by members of the platform. As a manager from industry noted: “Organization A through Z contributed arguments numbered one through infinity, and only some of these have succeeded.” We identified seven diagnostic frames in the data, and also three mechanisms which correlate with the micro-processes of framing (Gray et al., (2015): frame merging, importing a master frame and maintaining frame plurality.

We now describe each of the frames found in the case of the Nutrient Platform and also track how these frames were used throughout the three phases of the XSP’s development.

#### Frame 1: Environmental Impact of Phosphorus Use

This frame highlights the negative environmental impact of the current mode of using phosphorus and the damage that the nutrients in fertilizer cause to the environment.

Phosphorus goes in at one end and comes out at the other. And that will be a place where actually you don’t want to have it. It causes all kinds of problems. Eutrophication and waste, so that you will have to deal with that next. (Senior manager of a research institute)

Eutrophication is an issue highlighted in this frame. It refers to the extreme growth of plants and algae, especially in surface waters, that reduces water quality and biodiversity because other plants are crowded out: “The fertilizer ends up in the soil and then flushes out to the surface water. This causes an oversupply of nutrients, which in turn causes biodiversity to decrease. This means it is an environmental issue” (Second Secretary [coordinator] of Nutrient Platform). When frame 1 is used, *merging* is often used by platform members. Merging frames is defined as the construction of “a new frame from existing ones, yielding a wider, more-encompassing frame that supplants the original ones” (Gray et al. 2015, p. 129). Combining frame 1 with frame 2 highlights the negative environmental impact of acquiring phosphorus. This aids the “amplification” of the frame—that is, frames generated at the microlevel move to the meso- and macrolevels and become more diffused (Gray et al. 2015, p. 120).

#### Frame 2: Environmental Impact of Phosphorus Mining

This frame refers to the harmful environmental effects of mining phosphorus to create artificial fertilizer: “Currently contaminated phosphorus is coming to the Netherlands, even in the products” (Agricultural business manager). Uranium and cadmium are the source of this contamination. “More radioactivity is brought to the soil if you scatter with artificial fertilizer on it” (CEO, waste-processing sector). In a joint platform communication, frame 2 is often *merged* with frame 1 to create a framing category of environmental concerns about how phosphorus is extracted and used: “Pollution should be decreased (contamination of phosphorous rock) and sustainability is important” (Source: Internal agenda).

#### Frame 3: Scarcity

The focus in this frame is on the impending shortage of phosphorus as a natural resource. This argument has two parts. On the one hand, the use of phosphorus is increasing: “The entire world market will only grow due to an increasing population and changing diets. So, more and more phosphorus is necessary for raising agricultural productivity” (Senior government manager). On the other hand, the reserves of phosphate rock are declining:

Phosphate deposits however are finite with limited duration. Cordell (2008) mentions between 50–100 years. Steen (1998) estimated that at the time economically exploitable reserves could be depleted within 60–130 years” (NWP 2010).

This frame is often maintained together with other frames—i.e., there is frame *plurality*. Frame plurality “involves interactants managing or tolerating multiple meanings drawn from overlapping, conflicting, interstitial or otherwise unrelated field spaces in the interest of getting work done” (Gray et al. 2015, p. 130). This occurs, for example, when in documents created by the platform the scarcity frame is combined with frame 4 (Lack of security of supply): “With the growing world population and ensuing demand for food there is a growing need for phosphorus to produce artificial fertilizer. Phosphorus reserves however are only found in a few places, mostly in Morocco/the Western Sahara and China” (Nutrient Platform 2012). Maintaining frame plurality creates a certain ambiguity that “allows adherents of each frame to retain their preferred approach in the presence of the other” (Gray et al. 2015, p. 130).

#### Frame 4: Lack of Security of Supply

The lack of security of supply of phosphorus is emphasized in this frame:

Raw phosphorus is found in natural reserves in only a few countries (Morocco, US, China, Russia, etc.). The EU imports large quantities of raw phosphorus materials and has (almost) no reserves. The US has used up nearly all its reserves and has stopped exporting phosphate rock, while China has effectively stopped export by introducing a 200% export tax. As a result, Europe is to a large extent dependent on phosphorus from Morocco. (Source: Internal report on European Conference)

Within this frame, a parallel is sometimes drawn with the fossil fuel situation:

Comparable to fossil fuel also for phosphorus control of the resources is in the hands of a limited number of countries. Most of the known reserves are in Morocco, the VS and China. China however has put an export tariff on phosphate recently. (Netherlands Water Partnership 2010).

Here, a master frame is *imported*. This occurs when actors strive to achieve legitimacy by linking their frames to those of successful social movements. By likening the lack of security over access to phosphorus to that of fossil fuel scarcity, actors aim to emphasize the dangers of lacking control over a crucial resource for everyday life. Platform members recognize that people may be more familiar with fossil fuel reserves than with phosphorus reserves and so import this master frame to increase the legitimacy of frame 4.

#### Frame 5: Sanitation in Developing Countries

The sanitation frame focuses on the advantages of reusing human excreta directly in agriculture, especially in areas where the soil is low in phosphorus: “Especially for developing countries it is interesting to create self-sustaining areas in terms of energy and food security. For example, by connecting cities (human excreta) to arable land for P-recycling” (Source: Internal minutes). When extended, this frame also suggests that recycling in the form of “reuse of P in human excreta would decrease more and more if the current sanitation technology of the western world were to be adopted by developing countries” (Smit et al. 2009). In the formation stage of the XSP, this frame was strongly emphasized by XSP partners: “At this moment the phosphate issue has no real relevance here in the Netherlands, but in the developing world it certainly does and needs to be put high on the agenda” (Internal memo). Within this frame, we later observed frame *merging*, as it connects to frame 1: “More households connected to a sewer system will lead to increased losses toward the oceans sediments” (Smit et al. 2009). The sanitation frame also *merged* with frame 6: “Recovering (and selling) of nutrients will turn sanitation into a financially sustainable business” (Source: Internal minutes). The mechanism of *importing* a master frame was also used as actors related this frame to the popular trend of ecological thinking: “Ecological sanitation has to do with ecology and thinking in cycles and recovering waste” (manager of an NGO). The aim is to achieve legitimacy for the sanitation frame by relating it to a more general, popular frame.

#### Frame 6: Availability of Dutch Phosphorus (P)

In this frame, the opportunities for Dutch companies related to recycling phosphorus are highlighted: “The NP seeks to create policies and market conditions that stimulate sustainable nutrient use. It will build on the special position of the Netherlands, having nutrient surpluses at their disposal in a (future) world of shortage” (source: internal strategic plan). The argument here is that the Dutch surplus in phosphorus can be traded:

Since we have realized that a large shortage of phosphorus is developing in the world, opportunities have sprung up for companies and the government: to turn an expensive waste problem (food waste, manure, sewage water) into a profitable export product and to that end to connect different waste streams and return phosphorus to the cycle. (Source: Internal memo)

This frame is often *merged* with frame 7 to create a more general frame of opportunity:

The economic value added is not yet completely clear, but the expectation is that over the entire cycle partners can reduce costs by valorizing waste streams. In addition, this can create new jobs and increase exports (not only in terms of nutrients, but also in terms of knowledge, technology, etc.) (Source: Internal memo)

#### Frame 7: Dutch Strengths in Governance and Innovation

Like frame 6, this frame highlights the opportunities for Dutch actors that arise from phosphorus shortage, but it focusses more on applying current strengths. These include research skills in the water sector: “Knowledge institutes really want to give the Netherlands an important position as a country of knowledge. So, they want to pursue fundamental knowledge development” (CEO of a sludge-processing company). The opportunity to apply governance strengths is also highlighted: “Sustainability and a cycle approach are central in the Dutch governance and strategic developments. In national and regional governments, companies and NGO’s as well as knowledge institutes” (Source: Internal memo). As mentioned earlier, this frame is often merged with frame 6.

#### Other Frame Selection Mechanisms

In addition, the *merging* of frames takes place on a more aggregated level when frames 1–5 are grouped together as “crisis frames” and frames 6 (Dutch P Availability) and 7 (Dutch governance/innovative strengths) as “opportunity frames.” Actors in the platform appear to be consciously aware of the effects of framing. For example, several respondents emphasize that the opportunities presented by recycling phosphorus will be more fruitful than focussing on crisis: “[We as a society] should look at it [the need for phosphorus recycling] more as an opportunity (from the viewpoint of society, participation, economic environment) than as a threat” (Source: internal agenda). “The slogan ‘No P, No Life’ is too negative. (…)The project should be more focussed on the opportunities and the trade possibilities. Currently the approach is too much based on the threats” (Source: Internal minutes).

What is noticeable is that the seven different frames and their re-combinations used by actors involved in the Nutrient Platform are maintained in juxtaposition. However, some evolution can be seen in the use of different frames over time. For example, frame 5 (sanitation) is emphasized more in the initial stages: “The platform’s first secretary [coordinator] was part of the NWP, of the sanitation cluster. Aqua for All and WASTE [member NGOs] are also closely related to the Netherlands Water Partnership, they are also part of the ‘sanitation corner.’” (Senior manager at a research institute). However, at a certain point some partners became disgruntled with this frame:

One of the problems then was the waste sanitation story. That was approached from a very impertinent pedantic point of view that went ‘It is dirty in other countries and people should be washing their hands, people should do this, people should do that’. It did not go any further than that you invested money. If you invested a million, a million came out but it would never be more than that. Then came the start of recycling nutrients and energy that we considered to be a very interesting trajectory that we could help shape. (Senior government advisor)

It appears that, in the long run, the sanitation frame (frame 5) clashed with other frames so that maintaining frame plurality became problematic. This frame was later dropped from the set of active frames. However, it appears that frames that do not overtly clash are maintained:

By working well together, sharing knowledge and investing together in a smart way, the Netherlands can be the first country in the world to create a sustainable market for recycled phosphorus. With that, Dutch businesses—with the perspective of growing P scarcity in the world—as first-movers will be able to achieve a competitive advantage in the international phosphorus trade [frame 6]. In addition, government [frame 7], and businesses will be able to cut costs substantially by valorizing waste/manure into a useful and valuable resource [frame 3]. For society, this means that this economic opportunity solves, or at least decreases, a large environmental problem [frame 1 and 2]. (Source: Internal memo).

Thus, a more limited number of frames were selected.

### Frame Retention

We consider frames to have reached the *retention* stage when there is continued exchange of frames inside as well as outside the Nutrient Platform. First, frames are formulated collectively by the XSP as the members of the platform interact in meetings and in small focus groups based on a common interest or project. In later stages of the XSP life cycle, the (planned) activities of the Nutrient Platform shift to reflect the elimination of the sanitation frame (frame 5). Plans for international sanitation activity feature prominently on meeting agendas in the early stages of the XSP life cycle but are appear less frequently over time. In the mandated maintenance stage of the life cycle, the activities outlined by the platform were mainly national in scope—emphasizing frames 6 and 7, and were no longer aimed at changing international practices, which frame 5 would have implied. This highlights that there was a clear clash between frame 5 and other frames in use, and could be a reason why this frame was eventually removed from the set of active frames.

Frame retention also occurs as the opinions of external stakeholders of the platform appear to be incorporated in the frames used in communication by the platform. For example, in the formation stage of the XSP life cycle, intense cooperation with several government departments was important for members of the Nutrient Platform as they were trying to influence national regulation. At this stage, the innovative strengths of Dutch government and business (frame 7) are also emphasized in platform communication. For example, in a project description from the formation stage of the Nutrient Platform written in 2008, there is an emphasis not only on the scarcity of phosphorus but also on the fact that the Netherlands occupies a particularly privileged position regarding this. An internal memo outlines four reasons why this is so: “(1) Nutrient collection: The Netherlands can help by simply collecting nutrients; (2) Nutrient recycling: The Netherlands has an exceptional amount of knowledge when it comes to recycling nutrients from waste streams, both technologically and in terms of policy; (3) Water use: The Netherlands historically is a frontrunner when it comes to water management, including our policy constructions in relation to agriculture and (4) Food production: The Netherlands belongs to the global top when it comes to intensive farming, including lawmaking and regulations when it comes to environmental impact.” This demonstrates opportunistic framing by actors, where frame plurality allows them to pick and choose the most pertinent frame for each interaction.

Lastly, frame retention also occurs when the opinions of individual members can be seen to be directly reflected in platform frames. Members of the steering group also emphasize that the platform aims to incorporate the diverse opinions found among members:

There is a significance for everyone because everyone is dependent on phosphorus, but there is also a difference between them. For us it is an environmental issue and an economic opportunity, for others it is about security of supply. As a result, we frame it differently for each stakeholder. (Second secretary of the Nutrient Platform)

Thus, in different phases of the platform life cycle (formation, mandated maintenance and open maintenance), different frames were used; some were selected, others were deleted, and some evolved, recombined and persisted over time.

## Discussion

**Fig. 3:**
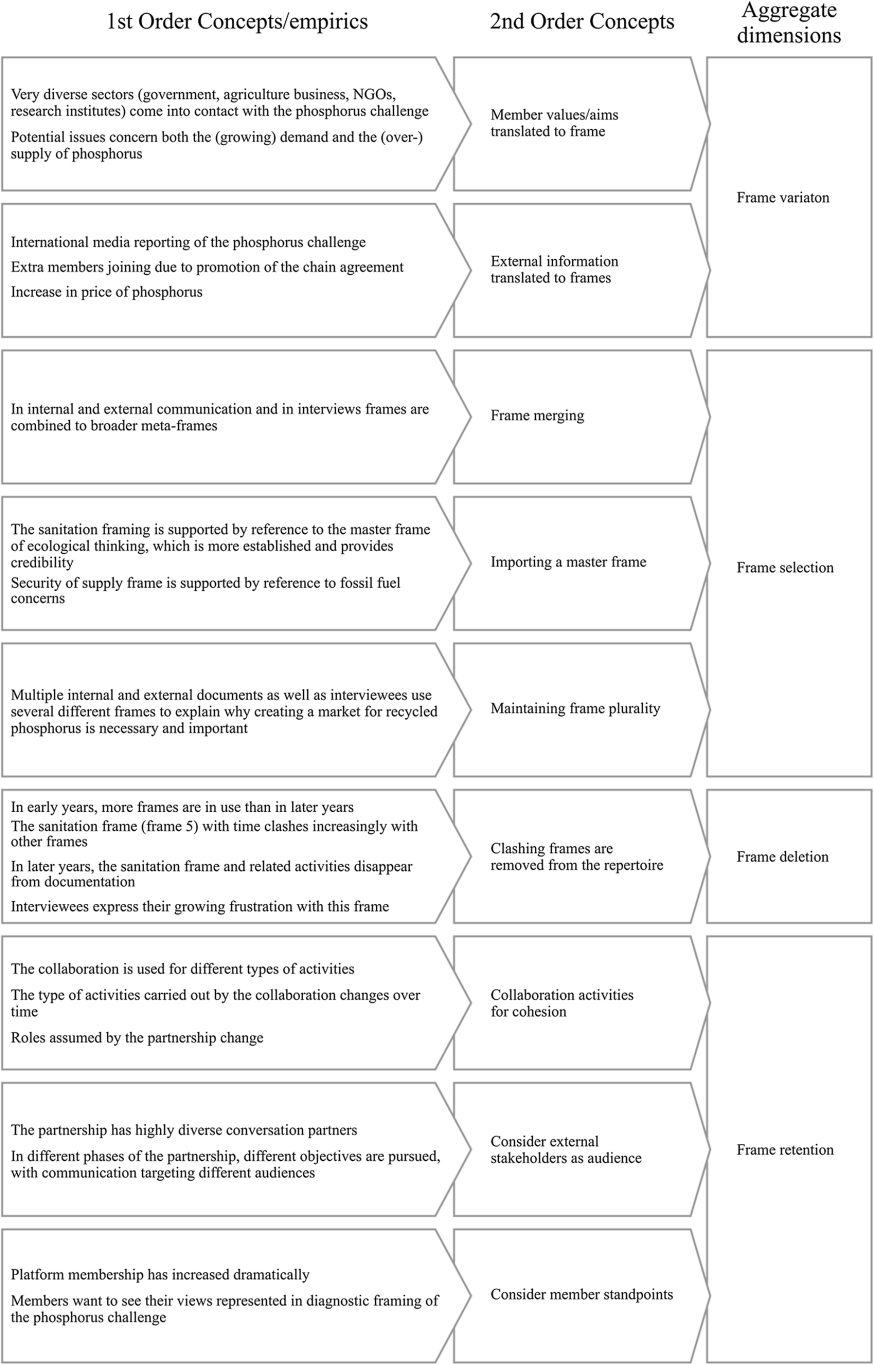
Data structure

We have assessed which challenges occur during the different life cycle phases of an XSP and how both strategic and interactional frames contribute to maintaining collaboration in an XSP. We have analyzed data over the first 8 years of existence of an XSP to identify how different frames come to exist in parallel and have explained the frame selection, deletion and retention mechanisms in use to achieve ongoing collaboration in an XSP. Our findings on the framing strategies for XSP maintenance correspond to the Variation–Selection–Retention model found in evolutionary theory (Aldrich 1999). Having multiple sources of frames causes variation, then selection, deletion and retention take place, resulting in a dynamic set of frames being in use at different times. The process is outlined schematically in Fig. 3 below.

### Frame Selection

Parties held different frames regarding how they viewed phosphorus. However, not all frames produced in interactions between platform members were selected for continued use. One example is the desire to extend scientific knowledge about the use of phosphorus, as mentioned by researcher from a knowledge institute. Many other frames could be traced to a few individual actors but never found their way into joint XSP communication. What the unselected frames had in common was that they were either very specific to a single party in the XSP, or they clashed directly with frames that were selected. An example is the desire to develop more international collaboration to incorporate expertise from other countries. This international collaboration frame clashed with frame 7, Dutch strengths in governance and innovation, and in the end, was not selected. As it turned out, an international phosphorus platform (the European Sustainable Phosphorus Platform) was launched some years later and became a rival of the Dutch Nutrient Platform in the competition for membership and funding. These examples suggest that the selection of frames delineates the direction and scope of an XSP and that selection occurs based on a desire for overall congruence around the key issue.

In their model of how cross-sector interactions move from contrast to fusion, Le Ber and Branzei (2010, p. 181) include the concept of *frame plasticity*, a process which involves the “effortful cycling back-and-forth between sector-specific, partnership-specific and organization-specific frames that allows the newly acquired understanding to fall into place for each of the partners.” Our results help to further clarify this process. We find that rather than moving toward a convergent frame, multiple frames continue to coexist, based on the notion of equifinality. Organizational members may have different reasons for undertaking action and different interpretations of the action’s potential outcomes, but they nonetheless act collectively to achieve the larger goal (Donnellon et al. 1986). In our case, members continued to adhere to their specific frames while agreeing on the final aim to recycle phosphorus.

From our coding, two factors emerged that affect frame selection: a motivation to achieve congruence between internal characteristics, which we refer to as *internal alignment*, and a motivation for congruence between internal and external characteristics, which we label as *external alignment*. Each of these can be further divided into two parts. We will specify these below.

#### Selection Through Internal Alignment

A drive to achieve *internal alignment* determines whether particular frames are selected. One aspect of internal alignment concerns the congruence between frames and XSP activities. Actors select frames that legitimate the platform’s activities. The platform was part-funded by the Dutch Ministry for Innovation and Environment, for example. As an active member of the XSP, the ministry promoted the initiation of real multi-party “business cases,” and thus, the frame of Dutch strengths in governance and innovation (frame 7) was selected. Conversely, we found that frame deletion occurs when day-to-day activities within the collaboration are not in accordance with a frame. In this case, it appears that practical difficulties of carrying out sanitation activities on another continent overshadowed the initial idea championed by NGOs. This resulted in the frame becoming redundant and eventually being discarded:

International Media Project: Not much progress. From now on we should put more effort in. The idea is to work out a broad media approach with different types of communication. A Terms of Reference document is ready, a partner should be found for co-financing. (Internal progress report, June 2012)

Our findings suggest that, at times, pragmatic considerations shape discussion topics and the frames that are selected. However, the chronology of internal documents also suggests that a frame can be deleted by a process of persistent ignoring and disuse, rather than because of overt conflict.

In addition to internal alignment based on XSP activities, frame selection is also influenced by *majority member frames*—i.e., the standpoint of most members. In the XSP’s initial years, frames relating to the scarcity and security of supply (frames 3 and 4) were emphasized:

As you know the Nutrient Platform is a network that aims to create possibilities for sustainable use of nutrients (…). Mainly driven by the impending shortage of phosphorous we aim to increase the speed of the transition towards sustainable nutrient use (Letter to invite new members to the platform, February 2011).

**Fig. 4:**
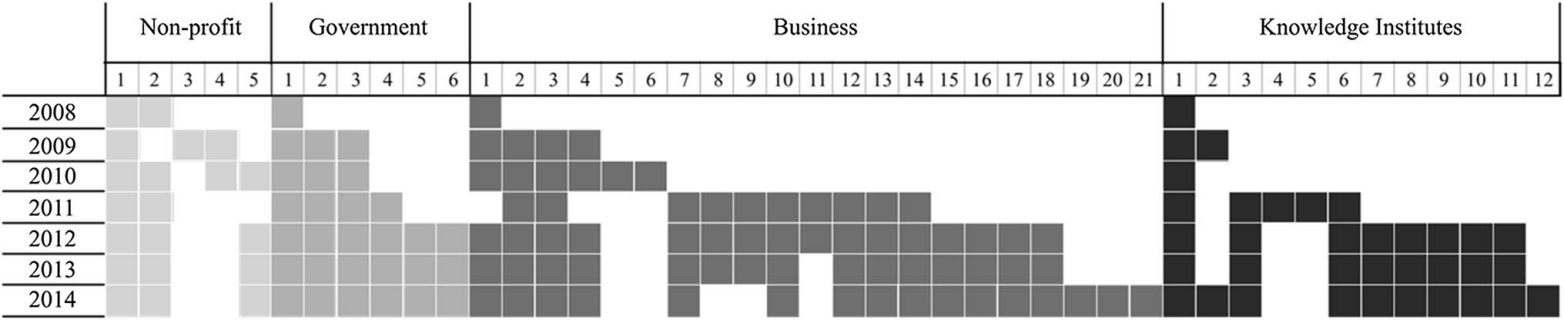
Nutrient Platform membership development. *One bar for each organization (a white square means no membership in a given year)

**Fig. 5:**
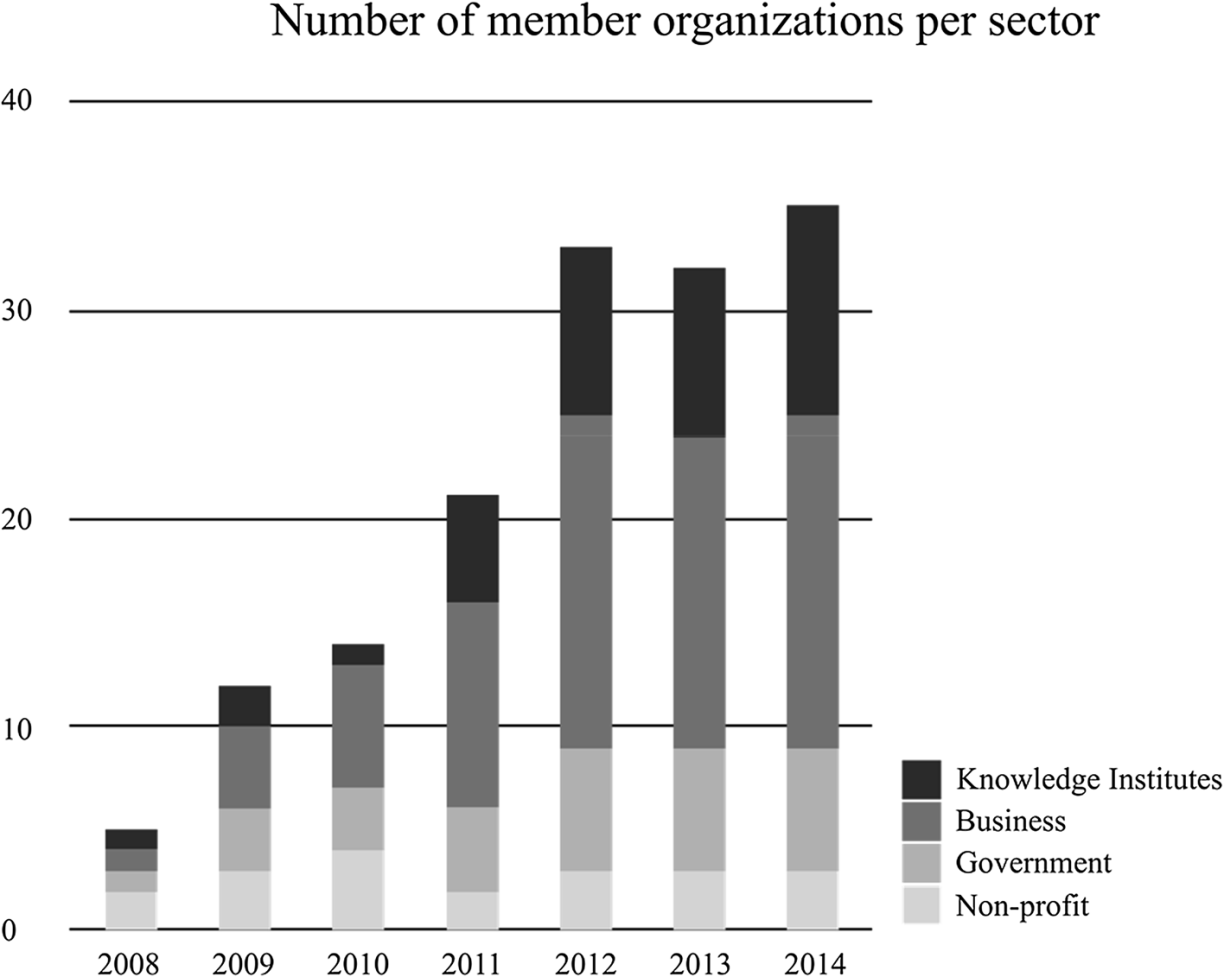
Nutrient Platform membership share per sector

As the Nutrient Platform expanded over time, more members joined, most of whom were *private sector* (business) partners, though there were also some research institutes (see Figs. 4, 5).

This meant that, as the platform’s member base changed, so did the frames agreed upon by most members. Simultaneously, in the later stages of the XSP’s existence, the two opportunity-related frames (frames 6 and 7) become more pronounced in platform members’ strategic choices and communication:

On January 21st the Nutrient Platform, together with the Dutch Embassy in Berlin, the Flemish Nutrient Platform and the German Phosphorus Platform, organized a successful symposium about the opportunities for phosphorus recycling in urban environments. (Nutrient Platform newsletter, February 2014).

This illustrates that the frames selected by the XSP were an apparent reaction to changing majority member frames or dominant frames.

On the other hand, the changing composition of the collaboration also meant that there was a drop in the number of NGO “focal interactants” (Gray et al. 2015), who largely supported a sanitation objective, (see Fig. 2), while the number of focal interactants with a more economic objective increased. This second group agreed that the sanitation frame (frame 5) was not a high priority:

In the beginning [other partners] also asked us to become involved in urine recycling in the south of Africa. Then we said, Listen, let’s first formulate a common goal. It is already an accomplishment if we become a club in the Netherlands, which reaches out to each other from the foundation of the problem to marketing and the solution. Let’s not start making it global, with lots of travel and writing of big documents, then nothing will happen. Then we become a talking club and there are already enough of those. (CEO of a fertilizer company)

The sanitation frame originated from NGO members of the platform. In the early stages of the platform NGOs were in the majority, with around a quarter of the total membership. However, by 2014 they represented less than ten percent of the membership (see Fig. 2), and the sanitation frame was purposefully deleted by most business and knowledge institute members, for whom other frames took precedence.

Our task has shifted since the first initiatives. There used to be quite a strong focus on sanitation, through the NGOs of course and… Well, I think there was a plan to do this in Africa but for now that has moved to the background. (Senior policy advisor for government)

The Nutrient Platform is a clear example of how politics and power differences “authorize certain actors and perspectives and neglect or exclude others” (Gray et al. 2015, p. 135, citing Meyer and Höllerer 2010). Overall, the process of aligning internally to the frames of the majority members within the platform establishes patterns of frame selection and deletion. We find that deletion can be a direct result of two things: persistent ignoring of certain frames plus pressure from a majority of partnership members.

#### Selection Through External Alignment

External factors also influence frame selection. There are two aspects to the motivation for external alignment. First, the *position of external stakeholders* appears to affect framing decisions. As mentioned earlier, the Nutrient Platform started with international and national aims, but in 2013 a spin-off—the European Sustainable Phosphorus Platform—was launched following a successful European Sustainable Phosphorus Conference. This organization became a partner but also something of a competitor as it was also seeking members:

I notice that I am moving more towards the European platform, because it is more useful for me than the Dutch Platform. Here I know most people now and I no longer need the platform to find them. (Senior manager from a research institute).

Since the emergence of this new stakeholder, the (Dutch) Nutrient Platform started placing more emphasis on the opportunity frames (frames 6 and 7) as these emphasize the advantages of the national platform over the pan-European version.

Second, we observed how *trends in public opinion* are used in frame selection to achieve external congruence and legitimacy. Members of the platform have since its foundation been very aware of public opinion and used the resulting momentum:

One of the things we thought of was that we should write an article to tell the public […] which we sent to the newspaper. […]A journalist then phoned me to plan a visit to interview me. […]Then for six or seven weeks I didn’t hear anything but one morning—I am subscribed to the same newspaper—I opened the paper and found that they made a front-page article out of it, titled “Food crisis due to phosphorus shortage”. […]He had used my original article but also interviewed other people. […]From that moment on people started approaching me and we formed the Nutrient Flow Task Group.” (Senior university researcher).

The scarcity frame (frame 3) became very pronounced in the media covering the issue. As a result, this frame was prominent during the Nutrient Platform’s early years of existence:

Next to the security of supply argument and the environmental argument there is also a scarcity aspect. This is used often in communication, as in ‘the supply of phosphorus is limited and when the mines are empty you will have a problem.’ (Second secretary [coordinator] of the Nutrient Platform)

Members of the platform aim to influence certain sectors of the public but the platform is also dependent on wider public opinion. Trends in public opinion may thus be leveraged by members in selecting frames that they believe will resonate best with an external audience.

### Frame Retention

After frames have been selected, actors seek to anchor them in the XSP using retention mechanisms. Burgelman (1991, p. 240) explains retention as “a form of organizational-level learning and distinctive competence, embodied in various ways—organizational goal definition, domain delineation, and shared views of organizational character.” In the Nutrient Platform, we identified several means by which the selected frames are retained.

Within the platform, the nature of activities carried out and tasks agreed upon in meetings changed over time, and the retention of frames appears to follow this pattern. For example, initially internal strategic documents and meetings were structured using three categories: international activities, European activities and national activities. Plans for each category were laid out and progress was discussed in meetings. In the European category, plans for a conference were quickly expanded, and in the national category, discussions about legislation gathered momentum. However, in the international category initiatives were not advanced and deadlines were postponed multiple times. Gradually, progress in the international section, which included work on sanitation projects in developing countries initiated by NGOs, stagnated, and less space and time were allotted to discussing this area of work. Coding from both internal documents and interviewees shows that the sanitation frame (frame 5) became less prominent as the concrete plans to improve sanitation in developing countries were pushed further down the agenda. Eventually the sanitation frame was abandoned. This suggests that the retention of active frames is related to the portfolio of XSP activities.

We identified two retention mechanisms. Frames are retained by promoting communication among the platform’s members, whereby selected frames are discussed and agreement on specific frames is emphasized. For example, notes from a members’ meeting (December 13, 2013) included the signing of a Memorandum of Understanding with the Flemish and German Nutrient Platforms. The notes state: “the signing of this document underlines the ambition of the Nutrient Platform to work with diverse actors in the value chain to create the right conditions for economically viable business cases around nutrient recycling.” This is an example of the retention of frame 6, in which the potential economic benefits of recycling phosphorus are emphasized.

Also, frames are retained by being enshrined in official documents that are shared with external stakeholders. Internally these documents are referred to and archived as “important final documents.” This facilitates retention by emphasizing the selected frames—as happened, for example, in the Phosphorus Chain Agreement, where members agreed to make a concerted effort to recycle phosphorus. Other examples include official letters sent to the Dutch government stating the aims and the progress of the Nutrient Platform. For example, in the official note sent in 2012 by the Nutrient Platform to the Dutch Lower Chamber, the scarcity frame (frame 3) is emphasized, as are the frames relating to the Dutch phosphorus surplus (frame 6) and the lack of supply security (frame 4). This also exemplifies how frames are retained in combinations and how the selection and retention of one frame does not exclude the possibility of another frame also being retained. In other words, it signifies ongoing frame plurality.

## A Model for Frame Plurality in XSPs

**Fig. 6:**
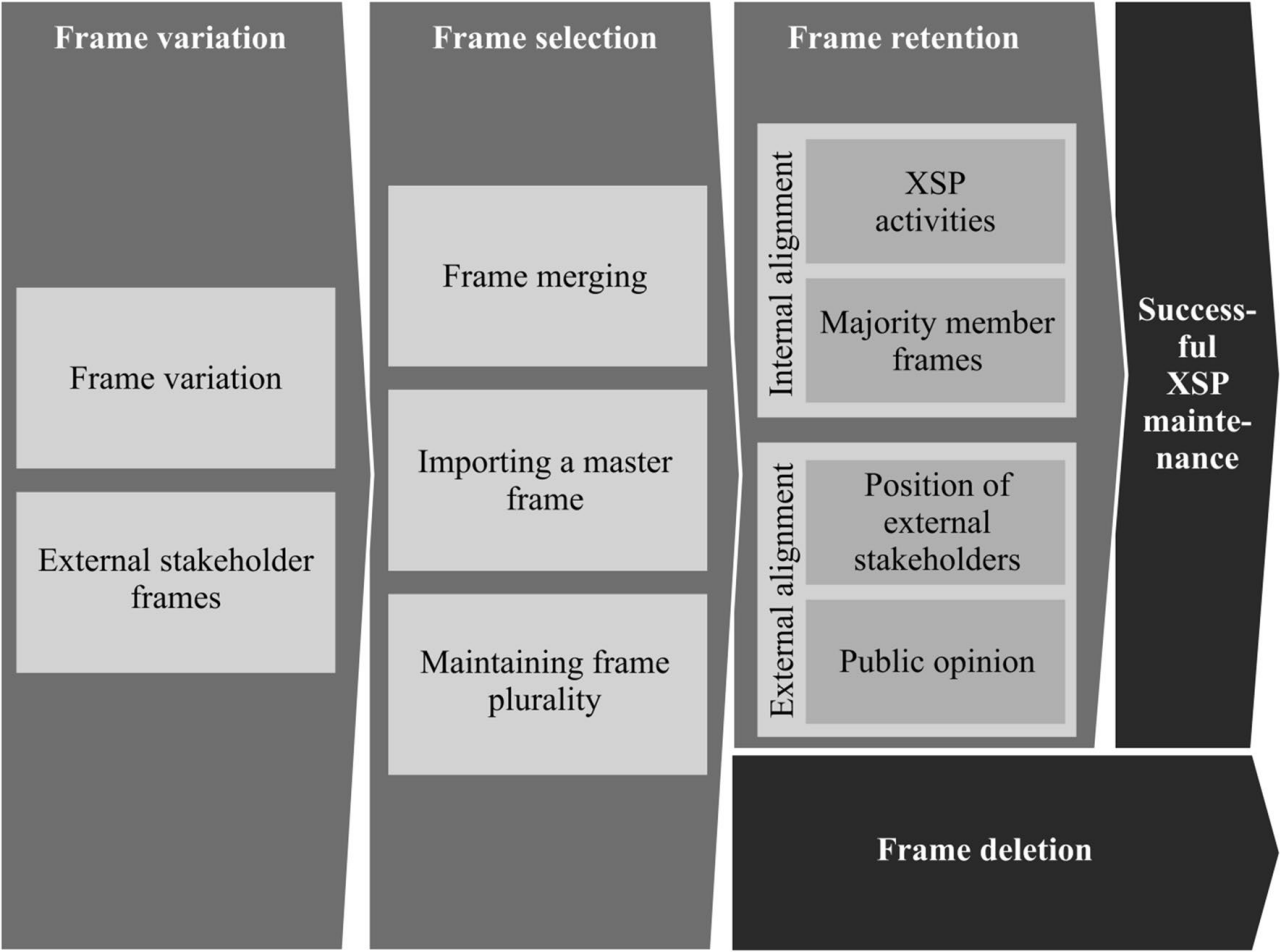
Model of frame variation, selection, retention and deletion for maintaining frame plurality

Drawing on the variation, selection, deletion and retention mechanisms described above, we develop a model for frame evolution leading to optimal frame plurality. See Fig. 6.

Frame variation in XSPs is caused by differing member standpoints or by information from external sources. The resulting frames are selected based on a legitimacy-driven desire for internal and external congruence as perceived by platform members. The selected frames are then retained through both internal interaction and external communication.

We find that, in the process of selection and retention, frame plurality is maintained. Frames that are at odds with each other can be maintained side by side, because there is agreement about the collaboration’s ultimate aim. Thus, valuable time and resources may be saved by avoiding the need for complete unanimity. However, frame plurality is not completely without bounds. Frames that are not (or are no longer) aligned with the majority point of view are deleted from the portfolio of active frames. We observed that as the sectoral composition of the XSP changed drastically, shifting the power equation, the sanitation frame was deleted. This suggests that, while maintaining frame plurality avoids the need for extensive discussion to enable XSP members to reach consensus over a single frame, the frames selected and retained in use are influenced by the power of the majority constituent.

By investigating the framing process throughout the different life cycle stages of an XSP, we bring a fresh perspective to the maintenance of collaboration in XSPs. Previous studies have suggested that collaboration efforts should be aimed at achieving an overall agreement through a convergence of the multiple frames used by different partners in a collaboration. Our case demonstrates that an alternative route to successful collaboration is to maintain a productive tension between the different frames. The result is that both internal and external stakeholders consider the collaboration to be acceptable, albeit for different motivations. Our analysis shows that sustaining collaboration in an XSP involves adapting frames in line with the changing institutional environment. Our model suggests that the constellation of frames that are actively used evolves over time, based on changing standpoints of members, changing demands of external stakeholders and changing collaborative activities.

In the variation stage in an XSP’s evolution, different frames produce variations in the meaning of the issue at stake. As more actors enter the platform, the number of frames increases. The process of selecting and discarding frames is not just a competitive process but also involves learning as people adapt to each other’s frames and identify commonalities and complementarities without necessarily giving up their own espoused frames. Over time, participants may tend to favor certain frames and avoid or ignore others. The move from variation to selection requires a frame to be less partisan, so that it can then appeal to a broader audience. While actors may push for their own positions, some frames become more comprehensible and acceptable over time, both inside and outside an organization (Strang and Meyer 1993). Thus, frames may not be selected “blindly” but through a more deliberate process based on learning and theorizing in ongoing interactions.

The selection of a few frames does not mean that these frames will become widely legitimated or institutionalized and thus retained (Gray et al. 2015). Plurality may involve some frames becoming dominant, enabling other non-dominant frames to continue if they have some evident link to these dominant frames. However, if a frame runs counter to the dominant frames or does not fit well to them (as was the case with the sanitation frame that was neither profitable nor seen to be in the national interest), it will then disappear. This process may be recursive in that the loss of a frame may in turn cause members who espouse that frame to look for alternatives and leave the XSP (e.g., the member who advocated internationalization became increasingly interested in the pan-European rather than the Dutch network). Thus, frames have a dynamic effect on the composition and recruitment of XSP members.

Only a few frames pass the selection hurdle, and even fewer are retained when they develop a collective meaning that goes beyond the platform and become “exteriorized” by both internal and external stakeholders. These frames may be developed internally within the XSP—majority member frames (e.g., national interest and business frames)—or linked with master frames imported from outside (e.g., the fossil fuel frame and the environmental frame). These retained frames may generate sufficient common ground among the platform’s participants to sustain collaboration and maintain the XSP even beyond its mandated maintenance stage, as in our case.

Our data suggest that once retention mechanisms are firmly in place, an XSP may remain relatively stable for a longer period of time. When all the parties involved can work with the plurality of frames in use, it appears that the typical pitfalls of an XSP (conflict and failure to create common ground) are mitigated. Parties may subscribe to only one or a few of the retained frames but still believe that their overall cause is being served and that the bigger issue at hand is still being addressed. On the other hand, the absence of any directly conflicting frames may help avert outright clashes between members.

In sum, maintaining optimal frame plurality can lead to and sustain collaboration among diverse participants because it allows multiple identities and interests to be accommodated simultaneously and does not force participants to converge around a single position. However, the plurality of frames needs to be manageable around an optimal number of retained frames. Excessive plurality may cause conflict between partners; this may potentially inhibit the emergence and sustenance of collaboration between diverse members and contribute to a failure to maintain the XSP. We find that frames are maintained in plurality when they are in congruence with XSP activities, majority member frames, the position of external stakeholders and the prevailing public opinion. Frames that do not meet these criteria may be deleted to avoid conflict and a reduction in the XSP’s overall effectiveness’. Striking a balance between too much and too little plurality may be key to sustaining collaborations such as XSPs.

### Contributions

*First*, while earlier studies have found that XSPs and multi-stakeholder partnerships go through different developmental stages (Gray 1985, 1989), we examine the process of evolution in an XSP and track changes in the frames used over time by the actors involved, looking also at the external and internal factors that coincide with these changes. By doing so, we add to studies that focus on the formation of an XSP and its developmental stages (Koschmann et al. 2012; Manning and Roessler 2014) or on XSP outcomes (Clarke and MacDonald 2016) by providing insights into the framing process through which collaboration may be sustained in an XSP after its formation.

*Second*, our notion of optimal frame plurality, while related to Le Ber and Branzei’s (2010, p. 164.) concept of frame fusion, also extends this work. Frame fusion—“the construction of a new and evolving prognostic frame and that motivates and disciplines partner’s cross-sector interactions while preserving their distinct contributions to value creation,” and the process of frame plasticity, where actors in organizations consciously select frames that fit with the partnership and the organizational and sector-related values. However, while Le Ber and Branzei (2010) focus on XSP dyads, we explain how optimal frame plurality is achieved among a vast array of diverse partners from different sectors in an XSP that changes in composition over time. In addition, we add further nuance to the notion of frame plurality (Gray et al. 2015) but show that plurality may have “finite” bounds as excessive variety may be counterproductive. We suggest that the deletion of certain frames, and the retention of a few—a progressively “narrowing frame bandwidth”—may be necessary for sustaining collaboration in XSPs. This is line with the argument by Patvardhan et al. (2015) that in complex inter-organizational settings (in this case an international consortium of “information schools”), it may be productive to seek to create “coherence” regarding shared problem domains, mutual interests, and practices, rather trying to reach absolute consensus through deliberation.

While we cannot support this argument with a counterfactual, our findings suggest that progress on agreements is thwarted by too many frames (excessive variety) and that the deletion of certain frames, and the retention of relatively fewer frames may be necessary for sustaining collaboration. We would be reluctant to put any definitive numbers on what is truly optimal in terms of frames as this is likely to considerably vary from one XSP to another, depending on the type of issue being addressed, the number and diversity of the parties involved and the external contextual influences. Thus, what is optimal may be situational and context-dependent. By optimal frame plurality, we refer to a level of variety in which diversity is neither smothered nor allowed to get out of hand, and which therefore allows a sufficient degree of agreement to emerge among the diverse constituents. Optimal frame plurality is thus not a definitive outcome but a continual balancing act that XSPs can consider aiming for in their efforts to reach a greater degree of consensus about how to address very complex social challenges.

Also, while not explicitly addressed in our study, our analysis suggests that framing happens in a politicized social context, and it matters *both who does the framing and what level of power and authority they have*—as was seen, for example, in how the changing composition of the XSP influenced the types of frame that became influential. Framing is thus inherently a “bidirectional” process (i.e., both top-down and bottom-up) (Gray et al. 2015), and the parties and the mechanisms available to them are both enabled and constrained by existing norms and power relations in any given setting.

*Third,* a rich body of work on hybrid logics and on hybridism more broadly has addressed how actors manage institutional plurality and complexity amid conflicting pressures from stakeholders. Such coping has been explained in terms of collective identity (Battilana and Dorado 2010; Patvardhan et al. 2015), identity aspirations (Kodeih and Greenwood 2014), selective decoupling (Pache and Santos 2013), selective synthesizing (Binder 2007; Chen and O’Mahony 2006) and temporal reflexivity (Reinecke and Ansari 2015). Studies have considered healthcare (Reay and Hinings 2005), social enterprises (Battilana and Dorado 2010), public-service partnerships (Jay 2013), biotechnology firms (Powell and Sandholtz 2012), universities (Murray 2010) and financial institutions (Smets et al. 2015). While this work has addressed both organizational and cross-sectional settings, the focus is on how actors manage plurality and collaboration on an individual basis by bridging, segmenting, recombining and reconciling frames across divergent stakeholder groups. We add to this work by explaining how plurality is managed *jointly* and how collaboration achieved by a collective in a cross-sector partnership comprised of diverse constituents. It is thus not so much what actors can do individually to manage conflict, but rather what they can do together that may matter more in an XSP.

## Limitations and Future Research Avenues

Although our study covers an 8-year period, our interview data was collected at the end of this period. We thus rely partly on retrospective accounts from interviewees. Fortunately, we could triangulate this information with rich archival data from earlier years. This proved to be especially helpful when studying the process of frame deletion. As this transpired to be a question of inaction rather than action, it would have been hard to uncover from interview data alone. Our access to data such as minutes and agendas has allowed us to study this process in detail. Future framing research could shed light on the hidden process of frame deletion by triangulating the “paper trail” of internal documents with interview data.

Another question is whether our findings are confined to collaborations in sectors that are heavily dependent on natural resources such as water, or whether they have wider implications. Given that this collaboration comprised a diverse mix of partners from engineering firms to government partners, we would argue that our findings are not strictly sector-specific. What is optimal, however, is likely to vary between different XSPs, depending on its characteristics, such as the type of issue, the number and diversity of parties involved, and the external contextual influences. Future research could investigate these dynamics in different contexts to shed more light on the claims we make. One could also ask whether our findings will hold true for collaborations with fewer or less diverse partners. Comparative research designs could examine the wider applicability of our findings.

## Conclusion

Based on our analysis of attempts to resolve a complex and at times controversial long-term social problem—namely dealing with the phosphorus challenge and achieving changes in both public perception and the regulatory environment—we offer a model of how actors in XSPs and multi-stakeholder partnerships achieve ongoing collaboration by maintaining an optimal level of frame plurality. Continual adaptation to internal and external factors results in the evolution of the set of frames used—through variation, selection, deletion and retention. We also find that concerted and sustained collaboration—a major challenge for most XSPs—does not have to result in a unanimous agreement around a single or convergent mega-frame; it can also emerge through generating productive tension between diverse positions and achieving optimal frame plurality and managed differentiation. In this way the integrity of the differing positions held by the various parties can be respected but sufficient common ground can still be found to allow collaboration on the complex issue at hand to be sustained. Optimal frame plurality is not a definitive outcome but rather an ongoing balancing act that XSPs can consider in their effort to foster greater convergence among diverse parties around highly complex social challenges.
